# A Validation Study of an Interviewer-Administered Short Food Frequency Questionnaire in Assessing Dietary Vitamin D and Calcium Intake in Swedish Children

**DOI:** 10.3390/nu9070682

**Published:** 2017-06-30

**Authors:** Lotta Söderberg, Torbjörn Lind, Pia Karlsland Åkeson, Ann-Kristin Sandström, Olle Hernell, Inger Öhlund

**Affiliations:** 1 Department of Clinical Sciences, Paediatrics, Lund University, SE-22100 Lund, Sweden; Lotta.Soderberg@skane.se (L.S.); pia.karlsland_akeson@med.lu.se (P.K.Å.); 2 Department of Clinical Sciences/Pediatrics, Umeå University, SE-90185 Umeå, Sweden; torbjorn.lind@umu.se (T.L.); Ann-Kristin.M.Sandstrom@vll.se (A.-K.S.); olle.hernell@umu.se (O.H.)

**Keywords:** dietary assessments, three-day food record, child, 25-hydroxy vitamin D

## Abstract

Vitamin D and calcium are essential nutrients with a range of biological effects of public health relevance. This study aimed to validate a short food frequency questionnaire (SFFQ) against a three-day food record (3D record), assessing the intake of vitamin D and calcium in Swedish children during wintertime. In a double-blinded, randomized food-based intervention study on the effect of feeding different daily doses of vitamin D supplement to 5–7-year-old children (*n* = 85), 79 (93%) participants completed SFFQ1 at baseline and SFFQ2 after the intervention, and 72 were informed to fill in a 3D record. The 28 (39%) children who completed the 3D record were included in this validation study. The baseline level of serum-25 hydroxy vitamin D [S-25(OH)D] was used as a biomarker. The correlation between all three instruments were moderate to strong. SFFQ2 and the 3D record correlated moderately to S-25(OH)D. Bland-Altman analysis showed that SFFQ2 overestimated vitamin D intake by on average 0.6 μg/day, (limits of agreement (LOA) 5.7 and −4.6 μg/day), whereas the intake of calcium was underestimated by on average 29 mg/day, (LOA 808 and −865 mg/day). Finally, the validity coefficient calculated for vitamin D using the method of triad was high (0.75). In conclusion, this SFFQ, assessed by a dietician, is a valid tool to assess dietary vitamin D and calcium intake in groups of young children.

## 1. Introduction

Vitamin D and calcium are two essential nutrients important for bone and dental health [1,2], and have a range of biological effects of public health relevance [3,4]. Vitamin D status is typically assessed as 25-hydroxy vitamin D [25(OH)D] in plasma or serum (S), as it reflects the sum of vitamin D from sunlight exposure and dietary sources [4,5]. Foods in general contain relatively minor amounts of vitamin D, the major sources being oily fish, egg, and vitamin D-fortified foods [6,7]. In Sweden, margarines and low fat milks are fortified with vitamin D_3_ [8] . Dietary sources of calcium are mainly dairy products [6,7]. In the Nordic Nutrition Recommendations (NNR 2012) [9] the vitamin D intake recommendation is 10 μg/day for children and the recommendation for calcium intake is 600 mg/day for children 2–5-years-old and 700 mg/day for children 5–9-years-old.

While calcium intake seems to be adequate in most children below 9 years of age [7,10], several studies denote low intake of vitamin D [7,10,11], resulting in insufficient vitamin D status [11,12,13,14,15,16,17] in this age group.

To assess the dietary intake of vitamin D and calcium, valid, reliable, and easy-to-use screening instruments are preferred both for clinical assessments and in scientific studies. The food diary or food record is one method to measure energy and nutrient intake in a valid and reliable way [18], and often described as “golden standard” [19]. Short food frequency questionnaires (SFFQs) are other valid screening instruments for estimating nutrient intakes if designed, validated [20], and used for specific groups [21], particularly if foods consumed on not a daily basis are to be captured. When judging and comparing calculated nutrient intakes, the methods uses to collect and calculate data should be comparable and validated [20]. Therefore, SFFQs used for calculating vitamin D and calcium intake should be validated, where many vitamin D-containing foods, which may not be consumed daily, can be captured.

The aim of this study was to validate the agreement of a new SFFQ for vitamin D and calcium intake during wintertime in young Swedish children with a three-day food record (3D record) with a hypothesis of good agreement and correlation, also using the method of triads.

## 2. Methods and Subjects

### 2.1. Study Design

This validation study was part of a longitudinal, double-blinded, randomized food-based intervention study on the effect of feeding different daily doses of vitamin D supplement to 5–7-year-old children. The study was conducted during late fall and winter (November 2012 to March 2013) when there is only minor sunshine and cutaneous synthesis of vitamin D is unlikely (“wintertime”). Children were recruited from well-baby clinics and school healthcare programs.

Inclusion criteria to the food intervention study were the age of 5–7 years, and the absence of gastrointestinal disease or cow’s milk allergy. In total, 85 children living in the city of Umeå in northern Sweden (63° N) were included. Umeå is a university city with 120,000 inhabitants. Parents not fluent in Swedish were assisted by an interpreter at the study visits.

In this study, a newly developed SFFQ was used first at baseline (SFFQ1) before the vitamin D supplements were introduced, and again three months later at the follow-up after the intervention (SFFQ2). A questionnaire was used to gather information in sex, ethnicity, parent’s education, day-care and school attendance, data on outdoor time activity, use of sunscreen during sunny days or visits to sunnier destinations at substantially lower latitudes, as well as general health and socioeconomic data. If the child was to visit a sunny country during the study period, the parents were recommended to use sunscreens with high sun protection factor for their child. At follow-up, all parents sufficiently competent in Swedish writing were asked to fill in a 3D record to be completed during the following 2–3 weeks. To be able to compare the vitamin D and calcium intake in the two SFFQs and 3D record, only ordinary foods were calculated while the supplements of 10 μg and 25 μg or placebo used during the intervention were excluded in the validation protocol.

Children who did not complete the two SFFQs were excluded from this study. The group of children included in the validation study had completed the SFFQ1, SFFQ2, and the 3D record and were compared to participants in the group of non-completers, who only completed SFFQ1 and SFFQ2. Reasons for not completing the food record and two SFFQs were not asked for.

The study was approved by the Ethics committee of the Medical Faculty of Umeå University and registered at clinicaltrials.gov (identifier NCT01741324).

### 2.2. Dietary Assessment and Calculations

A research dietician assessed food intake with the interviewer-administered SFFQ at baseline and follow-up during study visits. A short food frequency questionnaire was also chosen to capture less commonly consumed foods with respect to vitamin D intake. The SFFQ included 16 food items known to be important sources of vitamin D and calcium among children in Sweden. The SFFQ is estimated to cover about 98% of dietary intake of vitamin D and 80% of calcium [7]. Ten number of frequencies of monthly, weekly, and daily food intake were recorded, ranging from never to once a month; one to three times per month; once; twice; three; or four to six times per week; once per day; twice per day; or four or more times per day during the last three months. The parent was responsible for reporting the food intake in cooperating with their child during the estimation of food intakes. This means that the parent stated how often, what, and how much the child ate, but on certain occasions asked questions to the child. These issues mainly concerned milk intake at school, the number and type of spread the child used in school, as well as portion sizes at school. Menus from schools were used to support the estimations of food frequency. Illustrations of glasses and portions sizes were used to estimate volumes of milks, sour milks, and portions of spreads, fish, meat, egg, milk-based cereals, pâté, black pudding, pancakes, and cheese. Pictures of different sources of dairy and spreads were also used. A pilot study of the SFFQ had been conducted prior to the final design.

In the 3D records, all foods and beverage of all meals during three days at home, school, or day-care were consecutively recorded and included one weekend-day. Food items and portions sizes were specified in common terms or by household measures for quantities. The description of food with brands and ingredients were noted. A research dietician gave instructions to the participants’ parents on how to fill in the 3D record. During schooldays or meals at day-care, the teacher was asked and informed by parents about how to fill in the consumed foods. The 3D record was sent by letter or handed over at a third visit to the study center within four weeks after completing the intervention.

The Swedish Food Database (version 2011-06-21) [8] was used for estimations of vitamin D and calcium intake from the SFFQs. The 3D record intake was calculated with the Dietist XP software program (version 3.1, Kost och Näringsdata AB, Stockholm), which also uses the food composition database of the Swedish Food Database [8]. To evaluate the accuracy of the calculated energy intake of the 3D records, two methods were used. First, mean energy intake was compared to reference intake for age in the NNR 2012 [9]; 1267 kcal/day (5.3 MJ) for children aged 2–5 years and 1649 kcal/day (6.9 MJ) for children aged 6–9 years, for groups of children with average activity level and a mean weight of 16.1 and 25.2 kg, respectively. Next, the ratio of energy intake (EI) to basal metabolic rate (BMR) was calculated and compared by the Goldberg cut-off [22,23], suggesting a value of EI:BMR of 1.6 to be valid.

### 2.3. Anthropometric Measures and Biochemical Analyses

Height to the nearest millimeter was measured using a Seca 264 digital scale portable stadiometer (Seca, Hamburg, Germany). Weight was measured to the nearest 100 g, using a TANITA BWB-800MA. Body Mass Index, BMI (kg/m^2^) was calculated and converted to BMI *z*-score using the AnthroPlus-programme (available at http://www.who.int/growthref/en/) for children and adolescents aged 5 to 19 years based on the World Health Organization (WHO) reference dataset 2007 [24]. Blood samples were taken in non-fasting state at baseline, before intervention supplements were introduced and at follow-up after three months. S-25(OH)D was analyzed by mass spectrometry on an API 4000 LC/MS/MS system (AB Sciex, Framingham, MA, USA) (CV 4–6%) at the Department of Clinical Chemistry, Skåne University Hospital, Malmö, Sweden.

### 2.4. Statistical Analyses

At baseline, the Mann Whitney U test was used to compare descriptive characteristics of the participants as well as their vitamin D and calcium intake. Chi^2^-test was used to compare sex, ethnicity, day-care and school attendance, and parents’ education between the validation group and the non-completers group.

We analyzed the validity of the SFFQ based on the participants that completed SFFQ1, SFFQ2, and the 3D record, i.e., the validation group. The highest priority was given to the comparison of the SFFQ2 to the 3D record because of their closeness in time

The mean intake (SD) of vitamin D and calcium in the SFFQ1, SFFQ2, and 3D records were calculated. Participating children were their own controls and the paired sample Related Samples Wilcoxon Signed Rank test was used comparing the mean difference between the methods. Correlations between the two methods and with the biomarker S-25(OH)D were assessed with Spearman’s rho. Agreement between the two dietary methods (SFFQ2 and 3D record) as well as limits of agreement, defined as the mean difference ±1.96 SD, were assessed using the Bland–Altman method [25]. In addition, the inter rater reliability was quantified using a two-way mixed absolute agreement intra-class correlation coefficient (ICC) with corresponding 95% confidence intervals (CI) to assess the agreement between SFFQ2 and 3D record, allowing for the computation of measurement error ($\frac{\mathrm{SD}\;\mathrm{of}\;\mathrm{differences}}{\surd 2}$) and error range (measurement error × 1.96). Finally, we calculated the validity coefficient for the vitamin D SFFQ2 using a method of triads. This technique, originally developed by Ocké and Kaaks [26] measures the correlation between an observed vitamin D intake using the SFFQ and the “true” dietary intake. The validity coefficient is calculated as follows:

$$SFFQ\;validity=\sqrt{\frac{rQR\times rQB}{rBR}}$$

where rQR is the correlation between the vitamin D intake using the SFFQ2 and the 3D record, rQB is the correlation between the vitamin D intake using the SFFQ2 and the biomarker S-25(OH)D, and rBR is the correlation between the 3D record and the biomarker S-25(OH)D.

All statistical analyses were performed by using SPSS Statics version 22 (SPSS Inc., Chicago, IL, USA).

The level of significance was set at *p* < 0.05.

## 3. Results

At follow-up, 79 (93%) out of the initial 85 children had completed the two SFFQs. Seven of the children had parents with insufficient knowledge in Swedish writing and were excluded from filling in the 3D record. Of the remaining 72 participating children, the 3D records were returned from 34 (48%). Six records were excluded due to missing data, resulting in 28 participating children (39%) in the validation group.

Characteristics of the validation group are shown in the Table 1.

Age, weight, height, BMI *z*-score, sex, and vitamin D status were normally distributed and no significant differences were found between the validation group (*n* = 28) and the group of non-completers (*n* = 51) (data not shown). There were no significant differences between the groups regarding sex distribution, ethnicity, and attendance to day care or school, or in the parental level of education.

In the validation group, mean (SD) energy intake according to the 3D record was 1674 ± 354 kcal (7.01 ± 1.48 MJ) per day. Calculating EI:BMR, the value of the physical activity level was 1.6 ± 0.32.

There was a significant correlation between the calculated mean intake of vitamin D from SFFQ1 and SFFQ2 as well as between the mean vitamin D intake from each one of the SFFQs and the 3D record. Similarly, mean calcium intake correlated significantly between SFFQ1 and SFFQ2 as well as between each one of the SFFQs and the 3D record. Mean vitamin D intake was higher in SFFQ1 than in SFFQ2 and the 3D record, whereas calcium intake was similar regardless of the method (Table 2).

Furthermore, when including all 79 participating children, SFFQ1 and SFFQ2 correlated significantly for both vitamin D and calcium intake (r = 0.44; *p* < 0.001 and r = 0.45 *p* < 0.001, respectively). Intake of vitamin D and calcium did not differ between the validation group and the non-completers in SFFQ1 or SFFQ2 (data not shown).

The biomarker S-25(OH)D correlated significantly with vitamin D intake in SFFQ2 and 3D records (r = 0.475, *p* = 0.012 and 0.460, *p* = 0.016, respectively), but not in SFFQ1.

**Figure 1:**
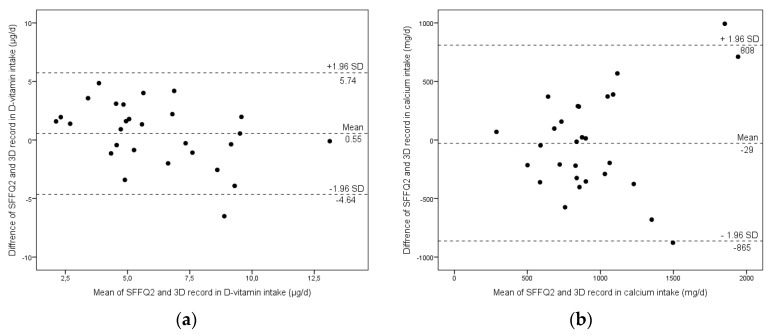
Bland-Altman plots show the mean difference and limit of agreement for vitamin D (**a**) and calcium (**b**) between short food frequency questionnaire 2 (SFFQ2) and the three-day food record (3D record) assessed in 28 5–7-year-old children from northern Sweden.

When compared with the 3D record (Bland-Altman analysis) (Figure 1), SFFQ2 overestimated the mean vitamin D intake by on average 0.6 μg/day (limits of agreement; 5.7 and −4.6 μg/day), whereas the intake of calcium was underestimated by on average 29 mg/day (limits of agreement; 808 and −865 mg/day).

For vitamin D, the SFFQ2 measurement error and error range was 1.87 μg/day and 3.67 μg/day, respectively; for calcium, the measurement error and error range was 591 mg/day and 1159 mg/day, respectively.

The ICC value of SFFQ2 and the 3D record for vitamin D and calcium were 0.52 (95% CI 0.30–0.72, *p* < 0.001) and 0.84 (95% CI 0.68–0.92, *p* < 0.001), respectively. Finally, the SFFQ2 validity coefficient was 0.75 [$\sqrt{0.538\times 0.475/0.460}$].

## 4. Discussion

### 4.1. Correlations between the Short Food Frequency Questionary, the Food Record and the Biomarker

This study shows that vitamin D and calcium intake in 5- to 7-year-old children, assessed with a new short food frequency questionnaire, correlates [27] well with a written three-day food record as well as with the biomarker S-25(OH)D. Since the study was conducted during late fall and winter when there is not efficient sunlight for vitamin D absorption, S-25(OH)D is a reliable biomarker of dietary vitamin intake in the method of triad. Several statistical approaches to evaluate the validity of the SFFQ confirm that the SFFQ is a valid measure to assess vitamin D and calcium intake in young Swedish children and may be preferred compared to conventional dietary food registrations, since the latter usually requires registrations over several days to correctly capture the vitamin D intake [20].

This SFFQ is to our knowledge the first one for comparative and combined analyses of vitamin D and calcium intake in young children, since previous FFQs have only been validated in adolescents and adults [19,28,29]. In children, FFQs have only been validated separately for calcium intake [30,31,32] or vitamin D intake in a long FFQ [33]. In addition, this is the first study using the biomarker S-25(OH)D to validate a food frequency in children using the method of triad to calculate the validity coefficient [27].

The SFFQ2 overestimated vitamin D intake by on average 0.6 μg/day, which is in accordance with expectations as it is difficult to correctly capture vitamin D intake from infrequently consumed vitamin D-rich foods in food records. Conversely, the intake of calcium was somewhat underestimated by on average 29 mg/day, which is of low clinical importance. The SFFQ2 correlated strongly to the 3D record for both vitamin D and calcium intake, which was also confirmed by the high ICC. This can be explained by their closeness in time, separated only by two weeks, while the SFFQ1 was completed three and a half months before the 3D record. In addition, repeated measures and trained knowledge to recall food portions and quantity may influence the results as suggested by others [28]. In the study of adolescent girls with anorexia nervosa and healthy controls, calcium and vitamin D intake were assessed with an FFQ and compared with a food record [28], and similar correlations were reported between the two methods for both vitamin D and calcium intake as in our study. Since S-25(OH)D correlated, although moderately, to the vitamin D intake, as assessed by both the SFFQ and 3D record, and the validity coefficient for vitamin D was high, and therefore the SFFQ seems to be a valid measurement tool for vitamin D intake in young Swedish children. In addition, there was a strong and significant correlation between SFFQ1 and SFFQ2 in the validation group as well as among the total of 79 children, supporting the repeatability of the SFFQ.

In a validation study of vitamin D intake in adults, where FFQ was compared to a four-day weight food record and S-25(OH)D, the mean difference and the 95% limits of agreement between the FFQ and food records were larger than in the present study; however, the validation coefficient was of similar magnitude [19].

### 4.2. Nutrition Intake and 3D Record

The 3D record did not underestimate the energy intake. To assess the accuracy of the 3D record, energy intake was first compared to reference values in the NNR 2012 [9]. The higher energy intake in the present study group may be explained by the higher body weight of the children in the present study compared to the reference data (NNR 2012) [9], and it is consistent with similar studies in young Swedish children [7,34,35]. Considering the low number of children in the present study, a value of 1.6 for energy intake in the 3D record, using the Goldberg cut-off for EI:BMR, is acceptable and reasonable [23]. Thus, energy intake of the 3D record in the present study is accurate and can be used to further validate the SFFQ.

Using food records to assess nutrient intake is considered more accurate and defined as the “golden standard” compared with other methods that rely on the memory [19]. On the other hand, the 3D record can underestimate more infrequently consumed but important vitamin D-rich foods, such as fatty fish [20,27]. Such underestimation of vitamin D, but not calcium, intake was also found in comparison with the SFFQs in the present study group. This most probably resulted from those important sources of vitamin D that were not captured during the three-day record, while calcium-containing foods such as dairy were consumed daily with little variation. The vitamin D intake assessed with the 3D record is in accordance with other surveys in Swedish children in the same age group. A national survey based on food records reported the intake of vitamin D to be 6.6 μg/day and 5.0 μg/day for 4-year-olds and 8-year-olds, respectively [7]. However, to capture vitamin D intake, the dietary assessment needs to cover a period of at least three months [20], and is thus only captured by using an SFFQ.

In accordance with others [7,12,13] and regardless of method, mean vitamin D intake was below the current Nordic recommendation of 10 μg/day [9], whereas the mean intake of calcium was higher [7,10,34].

### 4.3. Compliance Rate

Compliance was high to the SFFQ since they were filled out at the study visits, and assisted by a dietician. On the contrary, the completion rate of filling in the 3D record was low. In studies with both an FFQ and food record, it is common with lower compliance for the food record (71–94%) [28,31,32] which is higher compared to 39% in the present study group. A possible explanation is the fact that the participants did not meet the dietician at the third visit and that many parents forgot to send the food record by letter. Moreover, when day-care staff and school teachers needed to be involved, additional drops outs may have occurred. These data indicate that for most participants in the study group consisting of children in families with different origins, an SFFQ was easier to use.

### 4.4. Strengths and Limitations

The main strength of the study is that the biomarkers serum 25(OH)D and serum calcium confirm the dietary data, allowing us to calculate the validity coefficient using the method of triads. Another strength is the carefully collected food data that was obtained during a short time period and assisted by a dietician, and the calculations from a national database with reliable data on nutrients. A limitation is that less than half of the participants filled out the three-day food record, partly due problems with the Swedish language. However, by using the Bland and Altman method (25), the vast majority of measurements of vitamin D and calcium intake by the SFFQ and three-day record in the present study had sufficient agreement without signs of systematic bias, despite a limited sample size. This was further supported by a moderate to excellent ICC and a high validity coefficient. The strong correlations between SFFQ1 and SFFQ2 also support their repeatability.

Moreover, since there were no significant differences between families who completed the 3D record and those who did not regarding S-25(OH)D; dietary intake by SFFQ; anthropometrics; or socioeconomics, we suggest that our results can be representative for the group as a whole. Finally, the children were their own controls which further strengthens the results.

## 5. Conclusions

Dietary intake of vitamin D and calcium in young children as assessed with the new SFFQ correlated well with the conventional 3D record and with the biomarker serum 25(OH)D, and had good reproducibility. Since a larger number of children completed the two SFFQs compared to the 3D record (and the former method also captures less frequently consumed vitamin D-containing foods), this new SFFQ, validated for Swedish children aged 5–7 years, is a useful, repeatable, and reliable tool in surveys and dietary studies of children.

## Figures and Tables

**Table 1:** Baseline characteristics of 28 5–7-year-old children in a validation study of a short food frequency questionnaire assessing vitamin D and calcium intake.

**Status**	
Age, month (mean (SD))	74.8 (8.4)
Weight, kg (mean (SD))	24 (4.7)
Height, cm (mean (SD))	121.2 (7.8)
BMI ^1^, WHO ^2^ *z*-score (mean (SD))	0.39 (1.11)
S-25(OH) D, nmol/L (mean (SD))	58.0 (19.5)
**Sex, ethnicity, and parents education**	
Girls/boys (*n* (%))	14/14 (50/50)
Children born outside Sweden (*n* (%))	5 (18)
*Mother’s education level* (*n* (%))	
Primary school	5 (18)
Secondary school	4 (14)
Higher education	17 (61)
Other	2 (7)
*Father’s education level* (*n* (%))	
Primary school	1 (4)
Secondary school	1 (4)
Higher education	21 (75)
Other	5 (18)
*Children’s day-care* (*n* (%))	
Day-care centre	11 (39)
Pre-school	10 (36)
School	6 (21)
Other	1 (4)

^1^ Body Mass index; ^2^ World Health Organization.

**Table 2:** Comparison of the mean (SD) daily dietary intake and the correlation of vitamin D and calcium assessment between two short food frequency questionnaires (SFFQ1 and SFFQ2) and a three-day (3D) food record in 28 5–7-year-old children from northern Sweden.

Nutrient	SFFQ1	SFFQ2	3D Record	*p*-Value ^a^	Correlation ^b^ (*p*-Value)
Vitamin D, μg/day	7.29 (2.73)	6.42 (2.38)	5.87 (3.37)	^1^ 0.038	^1^ 0.707 (<0.001)
				^2^ 0.032	^2^ 0.418 (0.021)
				^3^ 0.172	^3^ 0.538 (0.003)
Calcium, mg/day	1066 (582)	930 (473)	959 (375)	^1^ 0.339	^1^ 0.490 (0.008)
				^2^ 0.339	^2^ 0.650 (<0.001)
				^3^ 0.663	^3^ 0.504 (0.006)

^a^ Related Samples Wilcoxon Signed Rand test; ^b^ Spearmans rho; ^1^ SFFQ1 versus SFFQ2; ^2^ SFFQ1 versus 3D record; ^3^ SFFQ2 versus 3D record.

## References

[1] Golden N.H., Abrams S.A. (2014). Optimizing bone health in children and adolescents. Pediatrics.

[2] Beto J.A. (2015). The role of calcium in human aging. Clin. Nutr. Res..

[3] Prentice A., Goldberg G.R., Schoenmakers I. (2008). Vitamin D across the lifecycle: Physiology and biomarkers. Am. J. Clin. Nutr..

[4] Holick M.F. (2012). The d-lightful vitamin d for child health. J. Parenter Enteral Nutr..

[5] Palermo N.E., Holick M.F. (2014). Vitamin D, bone health, and other health benefits in pediatric patients. J. Pediatr. Rehabil. Med..

[6] Eloranta A.M., Venalainen T., Soininen S., Jalkanen H., Kiiskinen S., Schwab U., Lakka T.A., Lindi V. (2016). Food sources of energy and nutrients in Finnish girls and boys 6–8 years of age—The PANIC study. Food Nutr. Res..

[7] Enghardt B., Pearson M., Becker W. (2006). Dietary Habits and Nutrient Intake in Swedish Children 4 Year Old and School Children in Grade 2 and 5 (Riksmaten—Barn 2003).

[8] Livsmedelsverket S. Livsmedelsdatabasen (Swedish Nutrient Data Base) 2011. http://www7.slv.se/Naringssok/.

[9] Nordic Nutrition Recommendations 2012: Integrating Nutrition and Physical Activity. http://dx.doi.org/10.6027/Nord2014-0022012.

[10] Ross A.C., Manson J.E., Abrams S.A., Aloia J.F., Brannon P.M., Clinton S.K., Durazo-Arvizu R.A., Gallagher J.C., Gallo R.L., Jones G. (2011). The 2011 report on dietary reference intakes for calcium and vitamin D from the Institute of Medicine: What clinicians need to know. J. Clin. Endocrinol. Metab..

[11] Ostergard M., Arnberg K., Michaelsen K.F., Madsen A.L., Krarup H., Trolle E., Molgaard C. (2011). Vitamin D status in infants: Relation to nutrition and season. Eur. J. Clin. Nutr..

[12] Öhlund I., Silfverdal S.A., Hernell O., Lind T. (2013). Serum 25-hydroxyvitamin D levels in preschool-age children in northern Sweden are inadequate after summer and diminish further during winter. J. Pediatr. Gastroenterol. Nutr..

[13] Persson K., Öhlund I., Nordström L., Winberg A., Rönmark E., West C.E. (2013). Vitamin D deficiency at the Arctic Circle—A study in food-allergic adolescents and controls. Acta Paediatr..

[14] Whiting S.J., Langlois K.A., Vatanparast H., Greene-Finestone L.S. (2011). The vitamin D status of Canadians relative to the 2011 Dietary Reference Intakes: An examination in children and adults with and without supplement use. Am. J. Clin. Nutr..

[15] Calvo M.S., Whiting S.J. (2006). Public health strategies to overcome barriers to optimal vitamin D status in populations with special needs. J. Nutr..

[16] Rockell J.E., Green T.J., Skeaff C.M., Whiting S.J., Taylor R.W., Williams S.M., Parnell W.R., Scragg R., Wilson N., Schaaf D. (2005). Season and ethnicity are determinants of serum 25-hydroxyvitamin D concentrations in New Zealand children aged 5–14 years. J. Nutr..

[17] Videhult F.K., Ohlund I., Hernell O., West C.E. (2016). Body mass but not vitamin D status is associated with bone mineral content and density in young school children in northern Sweden. Food Nutr. Res..

[18] Livingstone M.B., Robson P.J., Wallace J.M. (2004). Issues in dietary intake assessment of children and adolescents. Br. J. Nutr..

[19] Weir R.R., Carson E.L., Mulhern M.S., Laird E., Healy M., Pourshahidi L.K. (2016). Validation of a food frequency questionnaire to determine vitamin D intakes using the method of triads. J. Hum. Nutr. Diet..

[20] Millen A.E., Bodnar L.M. (2008). Vitamin D assessment in population-based studies: A review of the issues. Am. J. Clin. Nutr..

[21] Dickinson K.M., Delaney C.L., Allan R., Spark I., Miller M.D. (2015). Validation of a Brief Dietary Assessment Tool for Estimating Dietary EPA and DHA Intake in Australian Adults at Risk of Cardiovascular Disease. J. Am. Coll. Nutr..

[22] Goldberg G.R., Black A.E., Jebb S.A., Cole T.J., Murgatroyd P.R., Coward W.A., Prentice A.M. (1991). Critical evaluation of energy intake data using fundamental principles of energy physiology: 1. Derivation of cut-off limits to identify under-recording. Eur. J. Clin. Nutr..

[23] Black A.E. (2000). Critical evaluation of energy intake using the Goldberg cut-off for energy intake:basal metabolic rate. A practical guide to its calculation, use and limitations. Int. J. Obes. Relat. Metab. Disord..

[24] de Onis M., Onyango A.W., Borghi E., Siyam A., Nishida C., Siekmann J. (2007). Development of a WHO growth reference for school-aged children and adolescents. Bull. World Health Organ..

[25] Bland J.M., Altman D.G. (1986). Statistical methods for assessing agreement between two methods of clinical measurement. Lancet.

[26] Ocke M.C., Kaaks R.J. (1997). Biochemical markers as additional measurements in dietary validity studies: Application of the method of triads with examples from the European Prospective Investigation into Cancer and Nutrition. Am. J. Clin. Nutr..

[27] Itkonen S.T., Erkkola M., Skaffari E., Saaristo P., Saarnio E.M., Viljakainen H.T., Karkkainen M.U., Lamberg-Allardt C.J. (2016). Development and validation of an interview-administered FFQ for assessment of vitamin D and calcium intakes in Finnish women. Br. J. Nutr..

[28] Taylor C., Lamparello B., Kruczek K., Anderson E.J., Hubbard J., Misra M. (2009). Validation of a food frequency questionnaire for determining calcium and vitamin D intake by adolescent girls with anorexia nervosa. J. Am. Diet. Assoc..

[29] Park Y., Kim S.H., Lim Y.T., Ha Y.C., Chang J.S., Kim I.S., Min Y.K., Chung H.Y. (2013). Validation of a new food frequency questionnaire for assessment of calcium and vitamin D intake in Korean women. J. Bone Metab..

[30] Bertoli S., Petroni M.L., Pagliato E., Mora S., Weber G., Chiumello G., Testolin G. (2005). Validation of food frequency questionnaire for assessing dietary macronutrients and calcium intake in Italian children and adolescents. J. Pediatr. Gastroenterol. Nutr..

[31] Zemel B.S., Carey L.B., Paulhamus D.R., Stallings V.A., Ittenbach R.F. (2010). Quantifying calcium intake in school age children: Development and validation of the Calcium Counts! food frequency questionnaire. Am. J. Hum. Biol..

[32] Taylor R.W., Goulding A. (1998). Validation of a short food frequency questionnaire to assess calcium intake in children aged 3 to 6 years. Eur. J. Clin. Nutr..

[33] Nucci A.M., Russell C.S., Luo R., Ganji V., Olabopo F., Hopkins B., Holick M.F., Rajakumar K. (2013). The effectiveness of a short food frequency questionnaire in determining vitamin D intake in children. Dermato-Endocrinology.

[34] Garemo M., Lenner R.A., Strandvik B. (2007). Swedish pre-school children eat too much junk food and sucrose. Acta Paediatr..

[35] Sepp H., Lennernäs M., Pettersson R., Abrahamsson L. (2001). Children’s nutrient intake at preschool and at home. Acta Paediatr..

